# Enlarging Knowledge on Lager Beer Volatile Metabolites Using Multidimensional Gas Chromatography

**DOI:** 10.3390/foods9091276

**Published:** 2020-09-11

**Authors:** Cátia Martins, Tiago Brandão, Adelaide Almeida, Sílvia M. Rocha

**Affiliations:** 1 Departamento de Química & LAQV-REQUIMTE, Universidade de Aveiro, Campus Universitário Santiago, 3810-193 Aveiro, Portugal; catiamartins@ua.pt; 2 Super Bock Group, Rua do Mosteiro, 4465-703 Leça do Balio, Portugal; tiago.brandao@thebrowerscompany.com; 3 Departamento de Biologia & CESAM, Universidade de Aveiro, Campus Universitário Santiago, 3810-193 Aveiro, Portugal; aalmeida@ua.pt

**Keywords:** lager beer, volatile metabolites, foodomics, beer typing, HS-SPME, GC×GC-ToFMS

## Abstract

Foodomics, emergent field of metabolomics, has been applied to study food system processes, and it may be useful to understand sensorial food properties, among others, through foods metabolites profiling. Thus, as beer volatile components represent the major contributors for beer overall and peculiar aroma properties, this work intends to perform an in-depth profiling of lager beer volatile metabolites and to generate new data that may contribute for molecules’ identification, by using multidimensional gas chromatography. A set of lager beers were used as case-study, and 329 volatile metabolites were determined, distributed over 8 chemical families: acids, alcohols, esters, monoterpenic compounds, norisoprenoids, sesquiterpenic compounds, sulfur compounds, and volatile phenols. From these, 96 compounds are reported for the first time in the lager beer volatile composition. Around half of them were common to all beers under study. Clustering analysis allowed a beer typing according to production system: macro- and microbrewer beers. Monoterpenic and sesquiterpenic compounds were the chemical families that showed wide range of chemical structures, which may contribute for the samples’ peculiar aroma characteristics. In summary, as far as we know, this study presents the most in-depth lager beer volatile composition, which may be further used in several approaches, namely, in beer quality control, monitoring brewing steps, raw materials composition, among others.

## 1. Introduction

**Figure 1:**
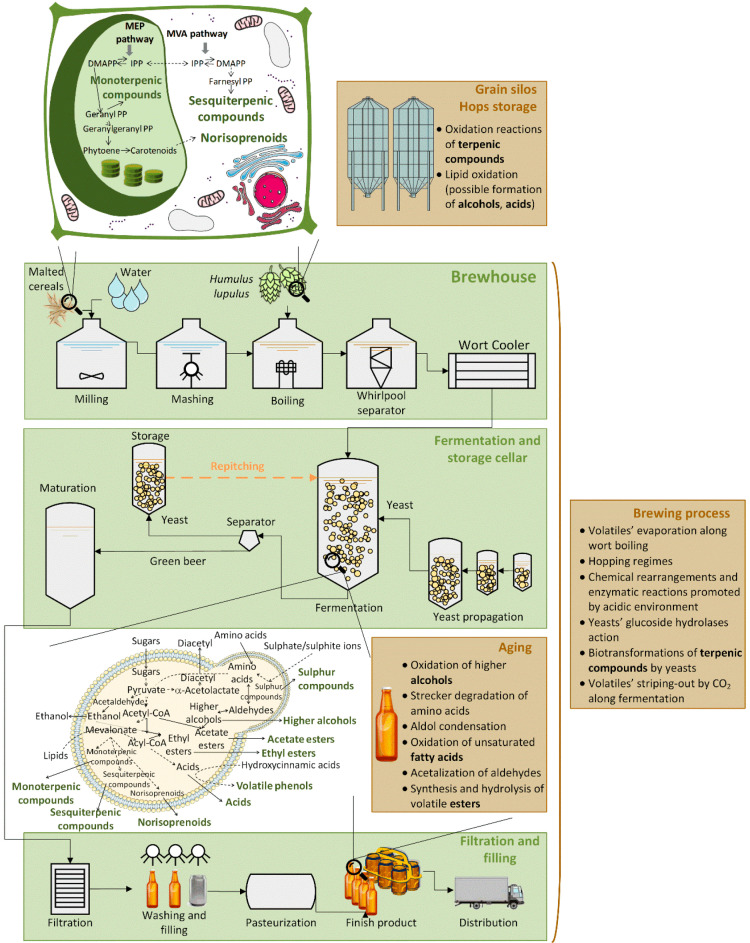
Schematic representation proposed to explain sources of the target analytes under study (acids, alcohols, esters, monoterpenic compounds, norisoprenoids, sesquiterpenic compounds, sulfur compounds, and volatile phenols), taking into account that they can be formed from raw materials and/or produced and biotransformed along brewing, also the main associated metabolic pathways were highlighted (magnifying glass) [3,4,5,6,7,8,9,10]. MEP pathway: methylerythritol 4-phosphate pathway; MVA pathway: mevalonate pathway.

Beer represents a broadly popular and widespread alcoholic beverage, and according to World Health Organization (WHO) statistics, it is the most consumed beverage type per capita in Europe (40.0%) and WHO Region of the Americas (53.8%) [1]. Hundreds of different beer brands are available in the market, being lager beers the main type to be produced and consumed worldwide [2], and consequently the most studied. Beer attractiveness arises from its pleasant organoleptic and nutritional attributes regarding a moderate consumption. Indeed, taste and flavor are the main factors which contribute for beer quality, thus conditioning the consumers’ acceptance. Overall and peculiar aroma properties of beer are dependent on its volatile components, whose origin and/or change can be attributed to several brewing steps, from raw materials until consumption. One particular source of these volatile components is mainly related with raw materials (cereals and hops) [3] and yeasts metabolism [4,5] (Figure 1). Plants secondary metabolites, such as terpenic compounds and norisoprenoids are the main chemical families from the raw materials that may be present in the beer metabolome. Moreover, the main yeast metabolites arise from diverse chemical families, such as acids, alcohols, esters, monoterpenic compounds, norisoprenoids, sesquiterpenic compounds, sulfur compounds, and volatile phenols [4,5]. Furthermore, chemical changes, which may occur in raw materials’ storage [6,7] and throughout the brewing process, as well as the phenomena of beer aging [8] may promote changes in the beer’s volatile composition.

Foodomics is an emergent field of metabolomics, which displays the profile of food metabolites and may assess food quality, safety, authenticity, or traceability. Food metabolome represents the gathering of small molecules (molecular weight lower than 2 kDa) called metabolites, that are present in foods and mostly derive from animals, plants, and microorganism’s metabolism. Moreover, food metabolites may be changed along food process, storage, microorganisms, or chemical contaminations. Each food has its own characteristics according to the presence and abundance of certain metabolites (which may be or not specific of one more origins) or even depending on the metabolites’ combination [11,12]. Thus, metabolomics might be helpful to comprehend the relation of the composition with the sensory and nutritional quality of foods. Metabolomics have been used to differentiate beers according to their type [13,14], cereals type [15,16], hops [17], or yeasts footprinting [18,19,20,21]. Moreover, beer volatile metabolites were tracked along the brewing process [22,23], different storage conditions [24,25], or dealcoholization process [26].

Foods volatile fraction gives functional information on sample-related variables (e.g., raw materials, processing or transformation technologies, storage conditions, etc.), and gas-phase extraction are good approaches to obtain an accurate chemical characterization. In this context, green solid-phase microextraction (SPME) combined with comprehensive two-dimensional gas chromatography with time-of-flight mass spectrometry (GC×GC-ToFMS) showed a huge potential to study the complex beer volatile composition [27]. In fact, the orthogonal analytes separation of GC×GC produces structured chromatograms, in which chemically-related analytes are distributed in a 2D “chemical map”, showing the diversity of the chemical structures of volatile and semi-volatile molecules [27]. This methodology was also applied to study the lager beer terpenic profiles, in which was achieved a beer terpen-typing according to producer system (macro- and microbrewers) [28]. Nevertheless, to go further to in-depth disclose beer aroma, several challenges have to be overcome in order to have high-quality and robust data, namely, the complexity and diversity of beer products, the chemical diversity of volatile and semi-volatile components, wide concentration dynamic range, difficulties of molecules identification, particularly due to the lack of standards, presence of unknow molecules, scarce databases from ToFMS, among others.

Considering the current state-of-the-art, the full potential of GC×GC for study the beer volatile composition is still far from being exploited. In fact, a detailed study of the lager beers’ chemical profiling can be obtained through GC×GC, studying an enlarged number of chemical families, for instance with aroma relevance. In this sense, this work intends to perform an in-depth profiling of lager beer volatile metabolites, using multidimensional gas chromatography. The acquisition of high-quality and robust GC×GC data will also allow to generate new chemical data to contribute to molecules’ identification, endeavouring to overcome the challenges presented previously. This goal was pursued with a set of 8 selected chemical families, namely, acids, alcohols, esters, monoterpenic compounds, norisoprenoids, sesquiterpenic compounds, sulfur compounds, and volatile phenols. Lager beers (from different countries, batches from the same brewery, breweries, aging times) were used as case-study.

## 2. Materials and Methods

The sampling, reporting of chemical analysis, and data pre-treatment, processing, and interpretation were performed according to the Metabolomics Standards Initiative (MSI) [29].

### 2.1. Materials and Reagents

Sodium hydroxide was supplied by Panreac (Barcelona, Spain). The retention index probe (an *n*-alkanes series of C_8_ to C_20_ straight-chain alkanes, in *n*-hexane) was purchased from Fluka (Buchs, Switzerland). The solid phase microextraction (SPME) holder for manual sampling and the fiber coating used were acquired from Supelco (Aldrich, Bellefonte, PA, USA), which included a 1 cm StableFlex™ fused silica fiber that was coated with partially cross-linked 65 μm polydimethylsiloxane/divinylbenzene (PDMS/DVB). According to the producer’s recommendations, the SPME fiber was initially conditioned at 250 °C for 30 min in the GC injector and daily for 10 min at 250 °C.

### 2.2. Samples

A total of 18 lager (Pilsner type) beers were analyzed in this study (Table 1):

- A total of 15 beers from 9 macrobreweries (P1–P9)—macro-bbs, which were available on the Portuguese market, from different countries (Portugal, France, Spain, and Germany) and batches, with alcohol contents from 4.2–5.2%, with shelf-life from 1–10 months;
- A total of 3 beers from 3 microbreweries (P10–P12)—micro-bbs, which were available on the Portuguese market, produced in Portugal, with alcohol contents from 5.0–5.5%, with shelf-life from 4–10 months.

### 2.3. Beer Volatile Metabolites’ Determination by HS-SPME/GC×GC-ToFMS

HS-SPME and GC×GC-ToFMS experimental parameters and decarbonation procedure were used as reported in a previous study [27] that was developed to characterize beer volatile composition. Summarily, 10 mL of beer were degassed overnight at 4 °C (static procedure) and then were placed into a 20 mL glass vial. Then, vial was capped and placed in a thermostated bath adjusted to 40.0 ± 0.1 °C, along with NaCl (2 g) and stirring bar (2 cm × 0.5 cm). The PDMS/DVB SPME fiber was inserted in the vial headspace for 30 min.

After the adsorption and absorption of beer volatile metabolites, the SPME fiber was manually introduced (30 s) into the LECO Pegasus 4D (LECO, St. Joseph, MI, USA) GC×GC-ToFMS injection port at 250 °C. This system consists of an Agilent GC 7890A GC (Agilent Technologies, Inc. Wilmington, DE, USA), with a dual stage jet cryogenic modulator (licensed from Zoex), a secondary oven, and a mass spectrometer with ToF analyzer. Equity-5 column (30 m × 0.32 mm I.D., 0.25 μm film thickness, Supelco, Inc., Bellefonte, PA, USA) was used as ^1^D column, and a DB-FFAP (0.79 m × 0.25 mm I.D., 0.25 μm film thickness, J&W Scientific Inc., Folsom, CA, USA) was used as a ^2^D column. The evaluation of the separation general quality and manual identification of peaks was performed through contour plots. For identification purposes, the mass spectrum and retention times (^1^D and ^2^D) of each component were compared to standards; the mass spectrum with those reported in mass spectral libraries, namely, in-house library of standards and two commercial databases (Wiley 275 and US National Institute of Science and Technology (NIST) V. 2.0–Mainlib and Replib). The identification was complemented by the experimentally determined linear retention index (RI) values through the use of van den Dool and Kratz equation [30]. RI determination was performed with the use of a C_8_-C_20_ *n*-alkanes series, whose values were compared with those reported in the bibliography for chromatographic columns similar to the above stated ^1^D column (Supplementary Table S1). The relative content of each volatile component in beer was estimated through the DTIC (Deconvoluted Total Ion Current) GC×GC area data that was expressed as arbitrary units (a. u.).

### 2.4. Statistical Analysis

Full data matrix consisted of 54 observations (18 beer samples, each one by three independent replicates) and 329 variables (peak areas of volatile metabolites). The list of all these volatile metabolites can be assessed in Supplementary Table S1, which besides the identification and chromatographic information also includes the GC chromatographic peak areas of the three independent replicates of each beer sample. Cytoscape v3.5.1 49 (The Cytoscape Consortium, San Diego, CA, USA) was used to build the systematization of 329 volatile metabolites detected in lager beer in study (Section 3.2), using the median of the GC peak area in all lager beer under study. The hierarchical cluster analysis (HCA) and heatmap was carried out using MetaboAnalyst 3.0 (web software, The Metabolomics Innovation Centre (TMIC), Edmonton, AB, Canada) [31]; data were autoscaled and normalized by the maximum, and the squared Euclidean distances and the Ward’s minimum variance as the clustering algorithm were used. Box plots were performed using the GraphPad Prism version 8 for Windows (trial version GraphPad Software, San Diego, CA, USA), and *t*-test was used to observe the significant statistical differences.

## 3. Results and Discussion

### 3.1. Chromatogram Contour Plot Analysis

In order to characterize the volatile metabolites present in beer, eight chemical families were selected according to the available literature [3,4,5] (Figure 1): acids, alcohols, esters, monoterpenic compounds, norisoprenoids, sesquiterpenic compounds, sulfur compounds, and volatile phenols. From these chemical families, monoterpenic and sesquiterpenic compounds were previously analyzed in our laboratory [28]. This previous targeted metabolomics approach [28] was focused on the in-depth coverage of beer terpenic compounds, in which a beer terpen-typing was achieved due to samples’ category clustering. Nevertheless, as this current study intends to characterize all the volatile metabolites present in lager beer, these chemical families (monoterpenic and sesquiterpenic compounds) could not be ignored, and therefore, they were also included in this study, being key elements of the beer volatile composition.

This targeted metabolomics approach, achieved by the high throughput and sensitive methodology based on HS-SPME/GC×GC-ToFMS, allowed the detection of 329 volatile metabolites from the 8 selected chemical families previously enumerated. As monoterpenic and sesquiterpenic compounds were previously reported [28], the remaining detected volatile metabolites were identified, being possible the putative identification of 181 volatile metabolites, from which 96 are reported for the first time in the lager beer volatile composition, which corresponds to an increase of ca. 53% of new chemical information. The putative metabolites’ identification was accomplished through a set of parameters that included the standards’ co-injection (when available), mass spectra comparison (home-made and commercial databases), calculation of retention index (RI_calc_) and their comparison with retention index from literature (RI_lit_) for columns of 5% phenylpolysilphenylene-siloxane (or equivalent), and analysis of the metabolites retention times according to the structured chromatogram principle (i.e., similar chemical structures are displayed in the same 2D chromatographic space, being a unique and distinctive criteria for metabolites identification by GC×GC-ToFMS) (Supplementary Table S1).

**Figure 2:**
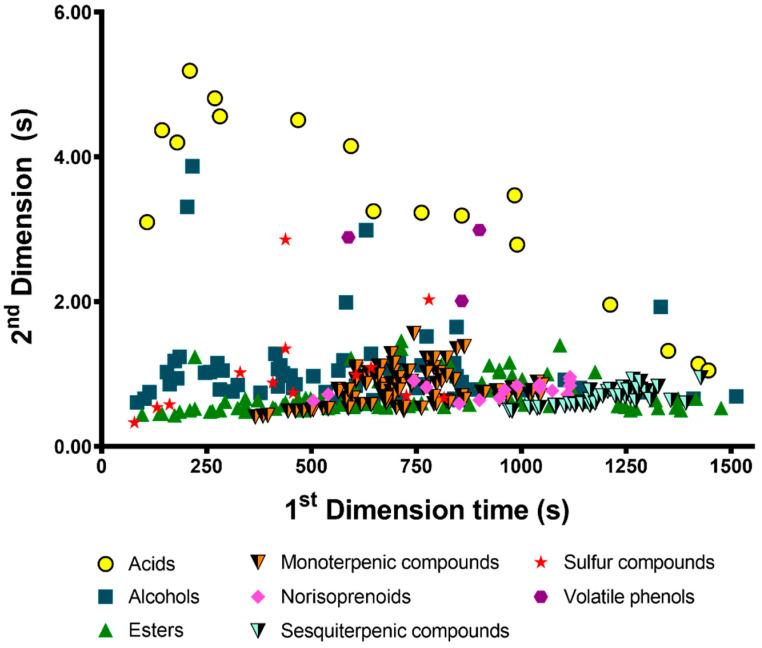
GC×GC peak apex plot with retention time coordinates of the 329 detected analytes in lager beer.

The distribution of the retention time’ coordinates of the 329 volatile metabolites is illustrated in the peak apex representation, which can be observed in Figure 2. This figure shows a practical example of the structured chromatogram, in which each metabolite is displayed in the chromatographic space according its physicochemical properties. Indeed, the orthogonal separation achieved by non-polar/polar (NP/P) set of columns that were employed, allows the volatile metabolites’ separation through their volatility (^1^D) and polarity (^2^D). For instance, esters tend to be the least polar compounds, presenting the global lower retention time for the second dimension (^2^*t*_R_); while acids, which present higher polarity, registered higher ^2^*t*_R_ value (Figure 2 and Supplementary Table S1).

The analytical variability of HS-SPME/GC×GC-ToFMS analysis was evaluated based on the data reproducibility, expressed through relative standard deviation (% RSD) of each analyte. DTIC GC×GC area was used to estimate its relative content. The % RSD of the detected volatile metabolites was examined, being achieved a median value of 20.9% for all metabolites, whereas the % RSD varied between 0.1% and 157%, minimum and maximum (Supplementary Table S1), respectively, considering each metabolite by itself. Furthermore, 52.4% of the peaks had % RSD lower than 20% (recommended limit for analytical variability of target bioanalysis by FDA [32]), and % RSD higher than 50% was only verified for 8.7% of the peaks, which indicated a good reproducibility.

**Figure 3:**
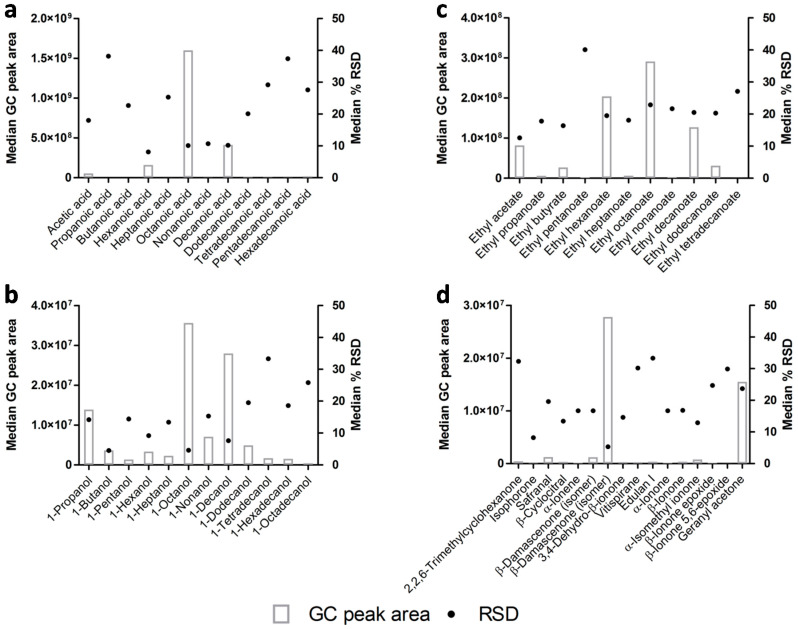
Analytical variability of HS-SPME/GC×GC-ToFMS analysis of beer volatile metabolites, considering the GC peak area (grey rectangle) and RSD (black circle) for selected homologous groups, namely (**a**) acids, (**b**) 1-alcohols, (**c**) ethyl esters, and (**d**) norisoprenoids. Volatile metabolites were displayed by increasing order of molecular weight on *x*-axis, except norisoprenoids that contain volatile metabolites with equal molecular weights.

Homologous groups from the same chemical family (that are structurally related) were selected to evaluate the data reproducibility related with the volatile metabolites’ molecular weight and also with respective GC peak areas. Acids, 1-alcohols, ethyl esters, and norisoprenoids were selected as examples (Figure 3). Figure 3 shows the % RSD (grey square) and respective GC peak area (black circle) of the previously mentioned volatile metabolites. Volatile metabolites were displayed in *x*-axis according to molecular weight (from lower to higher), only norisoprenoids presented volatile metabolites with equal molecular weights. In the case of acids (Figure 3a) and 1-alcohols (Figure 3b), there was observed higher RSD values for the volatile metabolites with higher molecular weight, possibly due to the increase of their hydrophobicity and more affinity with the matrix, which may perturb their extraction. For the esters, no relationship was observed between the variability of the data and the chemical characteristics of the molecules, namely the molecular weight (Figure 3c). Nonetheless, it is important to mention that it was possible to observe a relation between the % RSD and the detected GC peak areas, wherein lower RSD is observed for volatile metabolites with higher GC peak area (Figure 3), and several examples can be observed in Figure 3, for instance 10% for octanoic acid (Figure 3a), 5% for 1-octanol (Figure 3b), 8% for 1-decanol (Figure 3b), and 5% for β-damascenone (Figure 3d).

### 3.2. Profiling the Lager Beer Volatile Metabolites

The quality and trait of fermented foods, such as beer, can be monitored by metabolomics, e.g., evaluation of the changes of the metabolic profiles during fermentation and prediction of fermented foods quality, among others [33]. The comprehensive characterization of the beer volatile metabolites, a metabolite profiling strategy, was selected taking into account two potential sources of volatile metabolites in beer: raw materials (cereals and hops) and yeasts (Figure 1). In fact, monoterpenic and sesquiterpenic compounds may arise from raw materials, once they are reported to be synthetized through the mevalonate (MVA) and methylerythritol 4-phosphate (MEP, only expressed in plants metabolism) metabolic pathways [9]. Cereal and hops may also contribute to the presence of norisoprenoids in beer volatile composition, through the carotenoids’ degradation in plants [10]. Yeasts metabolism is the main responsible for the unique aroma profiles of beer, through the production of a wide range of volatile metabolites, particularly the target chemical families that were selected for this study: acids, alcohols, esters, monoterpenic compounds, norisoprenoids, sesquiterpenic compounds, and sulfur compounds. Indeed, they may arise from metabolism of carbohydrates and amino acids [5,10,34,35,36,37]; from biosynthesis of monoterpenic compounds, sesquiterpenic compounds, and fatty acids [38,39,40,41]; and also from de novo synthesis of sulfur compounds [34].

**Figure 4:**
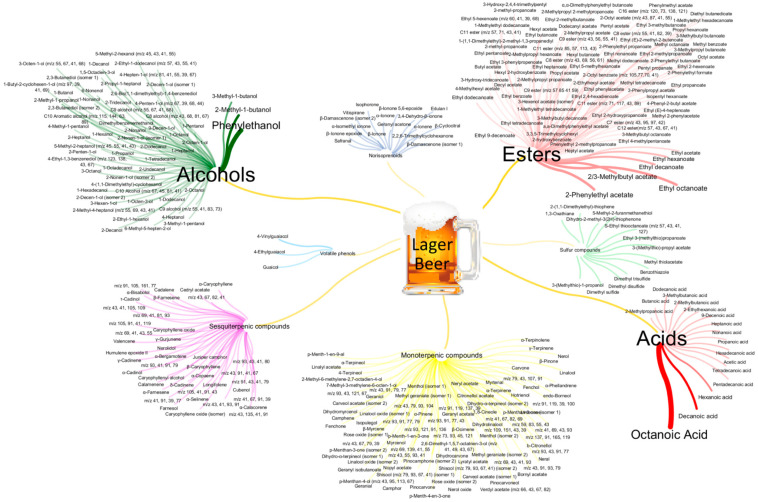
Systematization of 329 volatile metabolites detected in lager beer in study, distributed over 8 chemical families. Nodes correspond to chemical families and the target nodes are the detected metabolites. Edge thickness is linked to the amount (median GC peak area) of each metabolite, as well as the size of the nodes’ name.

The 329 detected volatile metabolites were systematized in Figure 4, in which the detected volatile metabolites (target nodes) were displayed and organized according to the respective chemical families (nodes). 

The chemical families with higher number of detected metabolites present in Figure 4 were esters and monoterpenic compounds (ca. 90 metabolites each), followed by alcohols (60), sesquiterpenic compounds (42), acids (17), norisoprenoids (16), sulfur compounds (13), and volatile phenols (3). Moreover, the amount of each metabolite (median GC chromatographic peak area in all lager beer under study) is reflected in the edge’ thickness, and also in the size of the volatile metabolites’ name (Figure 4), i.e., the higher GC chromatographic peak area is proportional to the highest volatile metabolites’ names and edge thickness (e.g., octanoic acid and phenylethanol were the volatile metabolites with the highest GC chromatographic peak area, considering all the lager beers under study).

The variety and diversity of the detected chemical structures from the 8 targeted families, even within each chemical family and also the wide range of detected amount (variation of GC chromatographic peak areas from 10^4^ to 10^9^) was only possible due to the methodology used. In fact, the orthogonal mechanism and ToF analyzer of GC×GC-ToFMS increases the chromatographic and spectral resolution and also the sensitivity, allowing the simultaneous analysis of major and trace analytes of beer within a single analysis.

From the detected volatile metabolites, it was possible to observe that 168 were present in all lager beers under study being called as common volatile metabolites. They were listed in Table 2 and represent ca. 51% of the total detected metabolites. An in-depth analysis of these 168 common metabolites allowed to construct Table 3, in which it is possible to compare the number and percentage of the common volatile metabolites within each chemical family. Table 3 shows that the number of common volatile metabolites varied between 22% and 100% from the total number of detected volatile metabolites depending on the chemical family. In fact, the chemical families can be ordered according to the increase of the percentage of common volatile metabolites: monoterpenic compounds < sesquiterpenic compounds < norisoprenoids < acids < alcohols < esters < sulfur compounds < volatile phenols. Therefore, the majority of monoterpenic and sesquiterpenic compounds were not present in all lager beers (only 22.0% and 33.3% of the total detected volatile metabolites, respectively), and they may contribute for the highest diversity and peculiar characteristics of the volatile composition of the lager beers under study. While volatile phenols and sulfur compounds were almost present in all lager beers (100% and 76.9% of the total detected volatile metabolites, respectively).

A set of 12 volatile metabolites were the major ones in lager beers that corresponded to ca. 81% of the total area of the targeted chemical families, namely, 3 acids—octanoic acid, decanoic acid, and hexanoic acid; 3 alcohols—phenylethanol, 2-methyl-1-butanol, and 3-methyl-1-butanol; and 6 esters—2-phenylethyl acetate, 2/3-methylbutyl acetate, ethyl octanoate, ethyl decanoate, ethyl hexanoate, and ethyl acetate. These 12 volatile metabolites were highlighted in bold in Table 2, which contributed highly to the highest total amount of the acids, alcohols, and esters. Indeed, all of these previously mentioned metabolites are frequently detected in beer volatile composition [46,50], being their main source related with yeast metabolism [55,56]. Moreover, these results are in agreement with those reported by Ruvalcaba et al. [49], which showed a similar set of metabolites that had the highest concentration in beers, namely, 3-methyl-1-butanol, ethyl octanoate, ethyl hexanoate, 3-methylbutyl acetate, 2-phenylethyl acetate, and octanoic acid.

The beer profiling can be helpful for the simultaneous screenings and/or follow-up of different aspects, such as the monitoring of the beer aroma (reported in Beer Flavor Wheel [57]) once these targeted chemical families have a huge impact on beer aroma properties, e.g., alcohol (n-propanol, 2-methyl-1-propanol), sour apple (ethyl hexanoate, ethyl octanoate), rose (phenylethanol, phenylethyl acetate), cheese (butanoic, hexanoic, octanoic acids), or fruity (β-damascenone, ethyl decanoate) aromas, among others. Furthermore, the detected volatile metabolites may be used to monitor the yeast metabolism [4,5,23,35], for instance the yeasts’ aminoacids uptake [35], e.g., uptake of leucine promotes the formation of 3-methyl butanol (one of the major volatile metabolites present in all lager beers under study). It also allows the detection of different off-flavors that may be monitored in the final product or along brewing process, namely, dimethyl sulfide (vegetables aroma, formed from malt during wort boiling) [58]; acids (cheese aromas) and sulfur-containing metabolites (putrid aromas), which can be associated to be produced by bacteria spoilage (e.g., *Pediococcus* spp., *Lactobacilli* spp., *Megasphaera* spp., *Pectinatus* spp.) [9,59]; 4-vinylguaiacol (clove-like aroma) that can be produced by wild yeasts spoilage (*Brettanomyces* spp., *Candida* spp., *Cryptococcus* spp., *Hansenula* spp., *Pichia* spp.) [9,59]; or β-damascenone (apple, peach, fruity aromas) that might be produced by carotenoid degradation or glycosides hydrolysis during aging [59].

### 3.3. Lager Beer Typing

**Figure 5:**
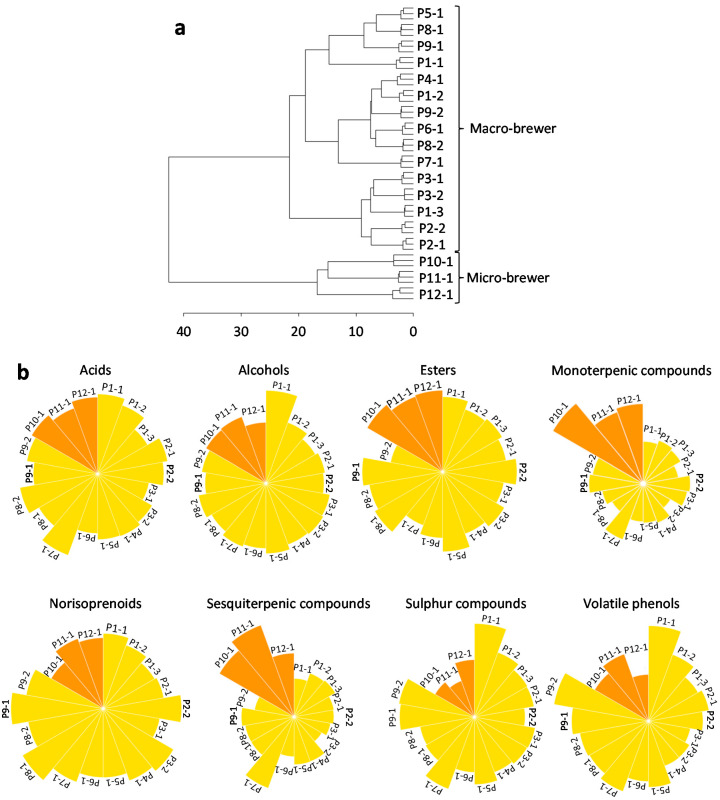
GC chromatographic data of the 329 detected volatile metabolites, from lager beers under study, were submitted to HCA (**a**), in which lager beers were grouped according to the production system: macrobrewer beers (P1–P9) and microbrewer beers (P10–12). The mean of GC chromatographic areas from each chemical family (**b**) is displayed for each lager beer (orange—microbrewer beers; yellow—macrobrewer beers).

A HCA was applied to the chromatographic data of the 329 detected volatile metabolites (data presented in Supplementary Table S1) from the lager beers under study, in order to characterize this data set by the clusters formation (natural grouping). The obtained dendrogram (Figure 5a) revealed the presence of two main clusters: commercial pasteurized macro-bbs and micro-bbs (including fresh and pasteurized beers). The detailed heatmap can be visualized in Figure S1. The production system was the main differentiating factor among the lager beers under study, and this type of clustering was previously reported only based on the monoterpenic and sesquiterpenic components [28].

It is noteworthy to perceive that each lager beer has its own specificities and inherent brewing process, and consequently, lower intra-variability among replicates (good clustering of the 3 replicates in Figure 5a and Figure S1) and higher inter-variability between the 18 lager beers under study from the 12 different producers was observed. Several factors contributed to the lager beers’ clustering, such as different raw materials, brewing (e.g., fermentation, clarification, filling, and pasteurization), and/or also the aging that varied for each lager beer under study. Moreover, as previously stated [28], microbreweries tend to have less brewing steps (e.g., clarification and possible stabilization of beer) and use different raw materials, these factors being possibly the main explanation for the clustering of the samples according to the producer system.

An in-depth analysis of the data was performed to understand the behavior of each chemical family among the lager beers under study. The mean of GC chromatographic areas of each chemical family from each lager beer under study is displayed in Figure 5b. Moreover, the chromatographic area of the common and non-common volatile analytes was also evaluated and compared considering the macro-bbs and micro-bbs, for each chemical family under study (Table 3 and Supplementary Figures S2–S12). Globally, micro-bbs have the highest GC chromatographic areas for most of the detected analytes (common and non-common analytes among all lager beers under study). The exceptions were observed for sulfur compounds that registered the highest GC chromatographic areas for macro-bbs (Figure 5b and Figure S8), and for common alcohols (Figure S3), common norisoprenoids (Figure S6), and common volatile phenols (Figure S8) there were not observed differences statistically significant between macro-bbs and micro-bbs. Giannetti et al. [45] showed different volatile profiles of industrial and craft Pilsner-type beers, particularly terpenic compounds and esters that had highest concentrations in craft beers, when compared with industrial beers, which are in agreement with our results.

Furthermore, as esters are important beer volatiles (have low thresholds and contribute for beers overall aroma [56]), a detailed analysis was performed for different ester classes, namely, short-chain (fruity aromas [16]), long-chain (oily aromas [16]), and acetate esters (fruity aromas [16]). Short-chain and long-chain esters showed highest GC chromatographic areas for micro-bbs (Figure S11), while acetate esters showed no statistical differences between macro-bbs and micro-bbs (Figure S12). Thus, acetate esters are not contributing for the differentiation of the esters profile between macro-bbs and micro-bbs.

Two main clusters according to production system (macro-bbs and micro-bbs) were only verified for alcohols and monoterpenic compounds (statistical analysis was performed to each chemical family, data not shown), being possibly that these two chemical families were the main contributors for the two main clusters that was observed when all the detected metabolites were used (Figure 5a and Figure S1). Furthermore, the global highest content of volatile metabolites was achieved for beer P1-1, particularly due to the higher relative peak area of 2-phenylethanol and 2-phenylethyl acetate (both volatile metabolites that may result from the yeasts’ degradation of phenylalanine [35] and also belong to the set of major volatile metabolites present in the lager beers under study), whereas the highest number of detected volatile metabolites was verified for beer P11-2 (Supplementary Table S1).

**Figure 6:**
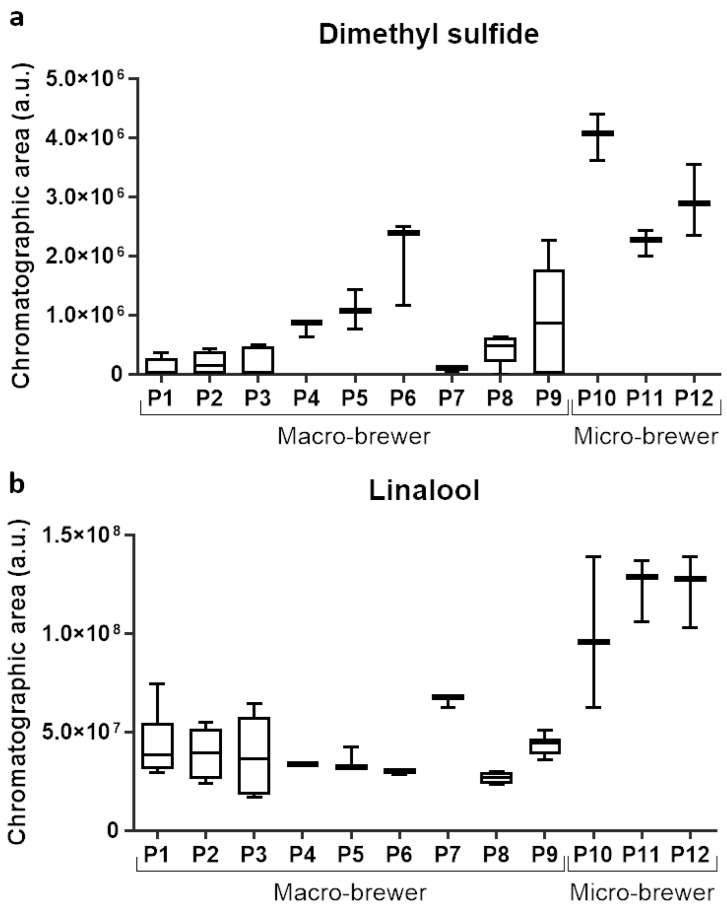
Box plots of the GC chromatographic area of specific metabolites, namely, dimethyl sulphide (**a**) and linalool (**b**), monitored in each beer producer under study: P1–P9 are macrobrewer, and P10–P12 are microbrewer.

This methodology can be also used to control and monitor the content of particular volatile metabolites, for instance that may be off-flavors or used for quality control. In this case, Figure 6a shows the amount of dimethyl sulfide in all samples under study, in which micro-bbs (P10–P12) had highest median GC chromatographic areas of dimethyl sulfide. This particular volatile metabolite is considered an off-flavor due to its vegetable aroma (if it is present in amount above its sensorial perception limit) and whose origin is reported to be from malt, and this volatile is used for the quality control of the wort boiling step [58]. These results show that it may not have such a good monitoring during wort boiling, which consequently may lead to higher contents of this compound in the lager micro-bbs under study.

Linalool, hop aroma indicator in beer [7], can also be monitored. The GC chromatographic area of linalool present in the lager beers under study is represented in Figure 6b, which showed that the median values for micro-bbs (P10–P12) were higher than macro-bbs (P1–P9). Micro-bbs employ different types of raw materials, for instance hop pellets, which when comparing with macro-bbs that use hop extracts, will influence the content of this analyte and may support this variation. A similar result was previously reported when comparing the linalool content between craft and industrial beers, it being higher for the first ones [45].

## 4. Conclusions

A comprehensive study of lager beer volatile composition was provided by profiling 329 volatile metabolites (wherein 181 were putatively identified, and 96 were reported for the first time), which were distributed over eight chemical families with potential impact on beer aroma, namely, acids (17), alcohols (60), esters (87), monoterpenic compounds (91), norisoprenoids (16), sesquiterpenic compounds (42), sulfur compounds (13), and volatile phenols (3). As far as we know, this study represents the most in-depth and detailed profiling of the lager beer volatile composition, which was only possible due to the combination of the extraction technique (SPME), which allows a direct analysis of beer without the addition of any organic solvent (fulfils the criteria for the green analytical chemistry techniques), with the high sensitivity, chromatographic resolution and high throughput technique, such as the GC×GC-ToFMS. The chromatographic data generated through the modulated chromatogram system can be further used as support of analytes’ identification. This detailed chemical profiling of lager beers allowed the simultaneous determination of a wide range of metabolites, namely, the major components such as acids, alcohols, and esters and the trace ones, like monoterpenic and sesquiterpenic compounds and norisoprenoids and sulfur compounds. Furthermore, data can be explored by univariate analysis to monitor, for instance, the content of target volatile metabolites, e.g., linalool and dimethyl sulfide. In fact, the combination of GC×GC-ToFMS with SPME may represent a useful tool for a streamlined evaluation of beer characteristics by constructing a multiple attribute methodology (MAM) workflow taking advantage of their sensitivity and high throughput attributes. According to the current green analytical chemistry concerns, such type of methodology that provides from a single analysis data from multi-analytes is preferred to methods using one analyte at a time.

Clustering analysis allowed a beer typing according to the beer production system: macro- and microbrewer beers. In fact, around half (ca. 51%) of the detected metabolites were common to all lager beers under study. Except for sulfur compounds, all the chemical families had the highest GC chromatographic areas for micro-bbs. Moreover, no statistical differences were observed for common alcohols, norisoprenoids, or volatile phenols between macro-bbs and micro-bbs. Monoterpenic and sesquiterpenic compounds were not present in all lager beers (only 22.0% and 33.3% of the total detected volatile metabolites, respectively), showing a wide range of chemical structures, which may give the beers unique characteristics. Furthermore, a set of 12 compounds (3 acids, 3 alcohols, and 6 esters) were the major volatile metabolites present in lager beers (ca. 81% of the total area of the targeted chemical families), their origin being associated to yeast fermentation, and they are also the main reported and monitored metabolites in beer volatile composition.

In summary, the generated data contributes to enlarge the knowledge on lager beer volatile metabolites that can be further applied and exploited to obtain relevant information in various contexts, such as the analysis of beer aroma (not only of lager beers but also of other beer styles) and subsequent used for their quality control, beer typing (e.g., understand similarities and differences between different beers, different brewing steps), monitor brewing steps (e.g., fermentation), and detection of off-flavors, among others.

## Figures and Tables

**Table 1:** List of lager beers analyzed in this study and respective available characteristics.

Sample	Category of Beer	Composition				Alcohol Content (%)	Shelf-Life at Analysis (Months)	Country of Production
		Malt	Unmalted Cereals	Hops	Others			
P1-1	Macrobrewer	Barley	Corn and barley	Extract	Glucose syrup	5.2	3	Portugal
P1-2	Macrobrewer	Barley	Corn and barley	Extract	Glucose syrup	5.2	5	Portugal
P1-3	Macrobrewer	Barley	Corn and barley	Extract	Glucose syrup	5.2	2	Portugal
P2-1	Macrobrewer	Barley	Maize or rice and barley	Extract	–	5.0	10	Portugal
P2-2	Macrobrewer	Barley	Maize or rice and barley	Extract	–	5.0	8	Portugal
P3-1	Macrobrewer	Barley	–	Extract	–	5.0	8	France
P3-2	Macrobrewer	Barley	–	Extract	–	5.0	5	France
P4-1	Macrobrewer	Barley	Maize and barley	Extract	E150c and E450	4.8	7	Portugal
P5-1	Macrobrewer	Barley	Corn and barley	Extract	–	5.1	3	Portugal
P6-1	Macrobrewer	Barley	Corn and barley	Extract	E150c and E405	5.0	1	Portugal
P7-1	Macrobrewer	Barley	Maize or rice and barley	Extract	–	4.2	9	Portugal
P8-1	Macrobrewer	Barley	Corn and barley	Extract	E150c and E405	5.0	3	Spain
P8-2	Macrobrewer	Barley	Corn and barley	Extract	E150c and E405	5.0	3	Spain
P9-1	Macrobrewer	Barley	–	Extract	–	5.0	6	Germany
P9-2	Macrobrewer	Barley	–	Extract	–	5.0	5	Germany
P10-1	Microbrewer (fresh)	Barley	–	Pellet	–	5.0	6	Portugal
P11-1	Microbrewer (fresh)	Barley	–	Pellet	–	5.5	4	Portugal
P12-1	Microbrewer (pasteurized)	Barley	–	Pellet	–	5.1	10	Portugal

Note: E150c—Ammonia caramel; E405—Propylene glycol alginate; E450—di-phosphates.

**Table 2:** List of the common volatile metabolites detected in all lager beers under study, using HS-SPME/GC×GC-ToFMS, including relevant chromatographic data used to assess compounds identification and those compounds that were previously reported on lager beer. More details, including chromatographic data, are available in Table S1. Bold names represent the major detected volatile metabolites.

^1^*t*_R_ (s) ^a^	^2^*t*_R_ (s) ^a^	Compound	CAS Number	MSI Level ^b^	RI _Calc_.^c^	RI _Lit_^d^	Previously Reported on Lager Beer
		Monoterpenic compounds					
606	0.91	Linalool	78-70-6	1	1101	1101	[28,42,43,44,45]
624	0.97	Fenchol	1632-73-1	2	1113	1118	[28,45,46]
636	1.02	Myrcenol	543-39-5	2	1122	1103	[28,47]
696	1.15	Borneol	507-70-0	1	1163	1166	[28,46]
708	0.95	Menthol	1490-04-6	1	1172	1174	[28]
714	0.86	(−)-Terpinen-4-ol	20126-76-5	1	1176	1180	[28,45,48]
732	1.04	α-Terpineol	98-55-5	1	1192	1195	[28,42]
774	1.22	7-Methyl-3-methylene-6-octen-1-ol	13066-51-8	2	1219	1222	[28]
792	1.09	(+)-β-Citronellol	106-22-9	1	1233	1234	[28,42,43,45,47]
792	1.22	Nerol	106-25-2	1	1233	1237	[28]
864	0.65	Bornyl acetate	76-49-3	2	1287	1288	[28]
912	0.74	Methyl geraniate (isomer)	2349-14-6	1	1323	1324	[28,47]
948	0.65	Citronellol acetate	150-84-5	2	1353	1354	[28,47]
966	0.70	Neryl acetate	141-12-8	2	1364	1362	[28,42]
444	0.49	β-Myrcene	123-35-3	1	988	988	[28,42,43,45,47]
582	0.67	Fenchone	7787-20-4	2	1084	1093	[28]
666	0.79	(1R)-(+)-Camphor	464-49-3	1	1142	1147	√ [28]
810	0.91	*p*-Menth-4-en-3-one	5113-66-6	2	1246	1251	√ [28]
618	0.58	Rose oxide (isomer)	16409-43-1	1	1109	1114	√ [28,46]
720	0.54	*m/z* 91, 119, 39, 100	–	3	1180	–	√ [28]
		Sesquiterpenic compounds					
1206	0.76	Nerolidol	7212-44-4	1	1571	1573	[28,43,47,49]
1212	0.79	Caryophyllenyl alcohol	–	2	1577	1569	[28,43,45]
1242	0.82	*m/z* 43, 67, 82, 41	–	3	1610	–	[28]
1260	0.68	Cubenol	21284-22-0	2	1634	1643	[28]
1272	0.75	τ-Cadinol	5937-11-1	2	1651	1651	[28,42]
1080	0.57	α-Caryophyllene (α-Humulene)	6753-98-6	1	1456	1456	[28,42,45]
1104	0.56	γ-Gurjunene	22567-17-5	2	1476	1472	[28]
1164	0.57	δ-Cadinene	483-76-1	2	1530	1524	[28,42,45]
1164	0.65	Calamenene	72937-55-4	2	1530	1528	[28,42]
1266	0.93	*m/z* 41, 67, 91, 39	–	3	1643	–	[28]
1164	0.78	*m/z* 91, 43, 41, 79	–	3	1530	–	[28]
1236	0.70	*m/z* 93, 43, 41, 80	–	3	1601	–	[28]
1308	0.65	*m/z* 43, 135, 41, 91	–	3	1701	–	[28]
1392	0.60	*m/z* 69, 41, 43, 55	–	3	1851	–	[28]
		Norisoprenoids					
504	0.63	2,2,6-Trimethylcyclo-hexanone	2408-37-9	2	1034	1051	–
744	0.91	Safranal	116-26-7	2	1197	1201	–
774	0.82	β-Cyclocitral	432-25-7	2	1219	1225	–
852	0.59	Vitispirane	65416-59-3	2	1278	1289	–
900	0.64	Edulan I	41678-29-9	2	1314	1314	–
960	0.78	β-Damascenone (isomer)	23726-93-4	2	1360	1383	[43,45,46,47,49,50,51]
990	0.84	β-Damascenone (isomer)	23726-93-4	2	1383	1383	[43,45,46,47,49,50,51]
1074	0.77	Geranyl acetone	689-67-8	1	1451	1455	[47]
1110	0.72	α-Isomethyl ionone	127-51-5	2	1481	1487	–
		Esters					
		Aliphatics					
96	0.44	**Ethyl acetate**	141-78-6	2	611	611	[23,42,46,47,50,52,53]
138	0.45	Ethyl propanoate	105-37-3	1	685	696	[50]
174	0.43	Ethyl 2-methylpropanoate	97-62-1	2	748	751	[51]
186	0.48	2-Methylpropyl acetate	110-19-0	2	769	769	[42,43,45,47,50,52,53]
210	0.50	Ethyl butanoate	105-54-4	1	806	806	[23,42,43,45,46,47,49,50,51,52,53]
222	0.52	Butyl acetate	123-86-4	2	816	819	[53]
222	1.24	Ethyl 2-hydroxypropanoate	97-64-3	2	817	819	[48]
258	0.48	Ethyl 2-methylbutanoate	7452-79-1	1	848	851	[52,53]
264	0.49	Ethyl 3-methylbutanoate	108-64-5	2	853	857	[43,49]
294	0.62	**2/3-Methylbutyl acetate**	123-92-2	2	879	877	[23,42,43,45,46,47,49,50,52,53]
324	0.53	Ethyl pentanoate	539-82-2	2	905	906	[47,49,51]
342	0.57	Pentyl acetate	628-63-7	2	917	916	[54]
342	0.66	C_7_ ester (*m/z* 43, 95, 97, 42)	-	3	917	-	–
372	0.64	Ethyl *(E)*-2-methyl-2-butenoate	5837-78-5	2	938	943	–
408	0.54	Ethyl 4-methylpentanoate	25415-67-2	2	963	967	[51]
414	0.52	Pentyl propanate	624-54-4	2	967	969	[54]
432	0.57	C_8_ ester (*m/z* 43, 69, 56, 61)	-	3	980	-	–
456	0.57	**Ethyl hexanoate**	123-66-0	1	996	996	[23,42,43,45,46,47,48,49,50,51,52,53]
480	0.59	Hexyl acetate	142-92-7	1	1013	1006	[23,43,45,46,47,48,49,50,53]
522	0.65	Ethyl 2-hexenoate	1552-67-6	2	1042	1045	–
552	0.55	Ethyl 5-methylhexanoate	10236-10-9	2	1063	1072	[45,47]
570	0.58	C_9_ ester (*m/z* 43, 56, 55, 41)	-	3	1075	-	–
600	0.55	Propyl hexanoate	626-77-7	2	1096	1101	–
600	0.57	Ethyl heptanoate	106-30-9	1	1096	1095	[23,43,45,48,50]
624	0.59	Heptyl acetate	112-06-1	2	1113	1113	[23,43,45,46,47,48,50]
642	0.60	Methyl octanoate	111-11-5	2	1125	1130	–
660	0.55	2-Octyl acetate (*m/z* 43, 87, 41, 55)	2051-50-5	3	1138	-	–
660	0.90	C_9_ ester (*m/z* 57 85 41 59)	-	3	1138	-	–
678	0.54	2-Methylpropyl hexanoate	105-79-3	2	1150	1154	–
678	0.56	2-Ethylhexyl acetate	103-09-3	2	1150	1159	–
744	0.59	**Ethyl octanoate**	106-32-1	1	1196	1196	[23,43,45,46,47,49,50,51,52,53,54]
762	0.60	Octyl acetate	112-14-1	2	1210	1211	[23,43,45,47,49]
876	0.58	Ethyl nonanoate	123-29-5	1	1296	1295	[43,45,47]
924	1.12	C_11_ ester (*m/z* 71, 117, 43, 89)	-	3	1333	-	–
948	1.03	C_11_ ester (*m/z* 57, 71, 43, 41)	-	3	1351	-	–
972	0.66	C_12_ ester (*m/z* 57, 43, 67, 41)	-	3	1369	-	–
978	0.93	3-Hydroxy-2,4,4-trimethylpentyl 2-methyl-propanoate	74367-34-3	2	1373	1364	[43]
996	0.64	Ethyl 9-decenoate	67233-91-4	2	1387	1388	[43,45,47,50]
1008	0.58	**Ethyl decanoate**	110-38-3	1	1396	1396	[23,42,43,45,46,48,49,50,52,53,54]
1068	0.56	3-Methylbutyl octanoate	2035-99-6	2	1446	1447	[43]
1164	0.62	Methyl dodecanoate	111-82-0	2	1530	1526	–
1176	1.03	3-Hydroxytridecanoate	107141-15-1	2	1542	1539	–
1230	0.56	Ethyl dodecanoate	106-33-2	2	1595	1594	[42,43,46,49,50,53]
1236	0.56	1-(1,1-Dimethylethyl)-2-methyl-1,3-propanediyl 2-methyl-propanoate	74381-40-1	2	1601	1607	–
1260	0.50	1-Methylethyl dodecanoate	10233-13-3	2	1634	1632	–
1296	0.78	Hexyl 2-hydroxybenzoate	6259-76-3	2	1684	1678	–
1326	0.53	Methyl tetradecanoate	124-10-7	2	1731	1726	–
1368	0.54	Ethyl tetradecanoate	124-06-1	2	1801	1801	[43]
1374	0.66	C_16_ ester (*m/z* 120, 73, 138, 121)	-	3	1814	-	–
1380	0.50	1-Methylethyl tetradecanoate	110-27-0	2	1826	1834	–
		Aromatics					
594	1.22	Methyl benzoate	93-58-3	2	1093	1096	–
702	1.07	Ethyl benzoate	93-89-0	2	1167	1172	[43,49]
714	1.35	Methyl 2-phenylacetate	101-41-7	2	1176	1179	–
714	1.46	2-Phenylethyl formate	104-62-1	2	1176	1174	–
810	1.13	Ethyl phenylacetate	101-97-3	1	1246	1248	[43,45,47]
828	1.15	**2-Phenylethyl acetate**	103-45-7	2	1260	1260	[23,42,43,45,46,47,48,49,50,51,52,53]
906	0.88	α,α-Dimethylphenylethyl acetate	151-05-3	2	1319	1320	–
948	1.06	2-Phenylethyl propanoate	122-70-3	2	1351	1357	–
972	1.16	3-Phenylpropyl acetate	122-72-5	2	1369	1388	–
1002	0.93	Phenylethyl 2-methylpropanoate	103-48-0	2	1392	1395	[46,49]
1062	1.00	2-Phenylethyl butanoate	103-52-6	2	1441	1441	[45,46,47]
1092	1.40	Ethyl 3-phenylpropenoate	103-36-6	2	1466	1480	[43,50]
1320	0.64	2-Octyl benzoate (*m/z* 105,77,70, 41)	6938-51-8	3	1721	-	–
1416	0.66	3,3,5-Trimethylcyclohexyl 2-hydroxybenzoate	118-56-9	2	1901	1904	–
		Acids					
108	3.10	Acetic acid	64-19-7	2	637	631	[23,43,45,47]
270	4.81	3-Methylbutanoic acid	503-74-2	2	862	861	[43,48]
468	4.51	**Hexanoic acid**	142-62-1	1	1007	997	[23,43,45,46,47,48,50]
762	3.23	**Octanoic Acid**	124-07-2	2	1212	1210	[23,43,45,46,47,48,49,50,53]
858	3.19	Nonanoic acid	112-05-0	2	1284	1288	[43,47,53]
990	2.79	**Decanoic acid**	334-48-5	2	1384	1387	[43,45,46,47,53]
1212	1.96	Dodecanoic acid	143-07-7	2	1578	1609	[43,53]
1350	1.32	Tetradecanoic acid	544-63-8	2	1772	1788	–
1422	1.14	Pentadecanoic acid	1002-84-2	1	1915	1878	–
1446	1.05	Hexadecanoic acid	57-10-3	1	1965	1964	–
		Alcohols					
		Aliphatics					
84	0.61	1-Propanol	71-23-8	2	591	589	[23,42]
102	0.66	2-Methyl-1-propanol	78-83-1	2	622	613	[23,42,43,45,46,47,50,51,52,53]
114	0.75	1-Butanol	71-36-3	2	643	639	–
156	1.03	**3-Methyl-1-butanol**	123-51-3	1	718	718	[23,42,43,45,46,47,48,49,50,51,52,53]
162	0.86	**2-Methyl-1-butanol**	137-32-6	2	728	728	[42,50,52]
174	1.18	4-Penten-1-ol (*m/z* 67, 39, 68, 44)	821-09-0	3	749	-	–
204	3.31	2,3-Butanediol (isomer)	513-85-9	2	803	796	[45]
216	3.87	2,3-Butanediol (isomer)	513-85-9	2	814	796	[45]
246	1.02	4-Methyl-1-pentanol	626-89-1	2	838	843	–
258	1.03	3-Methyl-1-pentanol	589-35-5	2	848	851	–
288	1.05	1-Hexanol	111-27-3	1	875	880	[23,43,45,46,47,48,49,50]
324	0.85	2-Heptanol	543-49-7	2	905	907	–
420	1.07	1-Heptanol	111-70-6	2	975	970	[43,45,46,47,48,50]
432	1.01	1-Octen-3-ol	3391-86-4	1	980	982	[45,52]
450	0.98	6-Methyl-5-hepten-2-ol	4630-06-2	2	996	996	–
456	0.81	3-Octanol	589-98-0	1	996	1001	–
462	0.86	2-Octanol	123-96-6	2	1001	1004	[46]
504	0.97	2-Ethyl-1-hexanol	104-76-7	2	1030	1034	[43,45,48]
558	0.63	2-Octen-1-ol	22104-78-5	2	1067	1074	–
564	1.05	1-Octanol	111-87-5	1	1072	1074	[23,43,45,46,47,48,49,50,53]
606	0.84	2-Nonanol	628-99-9	2	1101	1107	[43,45]
684	1.08	C_9_ alcohol (*m/z* 55, 67, 41, 68)	-	3	1159	-	–
702	0.63	2-Nonen-1-ol (isomer)	22104-79-6	2	1167	1151	–
708	1.00	1-Nonanol	143-08-8	2	1172	1174	[43]
714	0.64	2-Nonen-1-ol (isomer)	22104-79-6	2	1175	1179	–
840	1.15	9-Decen-1-ol	13019-22-2	2	1269	1272	[43]
846	0.97	1-Decanol	112-30-1	1	1274	1265	[43,45,46,47,49,50]
888	0.79	2-Undecanol	1653-30-1	1	1305	1309	[43,45]
1014	0.78	2-Dodecanol	10203-28-8	1	1401	1413	–
1104	0.92	1-Dodecanol	112-53-8	1	1476	1480	[43,47,48]
1410	0.66	1-Hexadecanol	36653-82-4	2	1889	1884	–
		Aromatics					
582	1.99	Dimethylbenzenemethanol	617-94-7	2	1085	1086	–
630	2.99	**Phenylethanol**	60-12-8	1	1119	1122	[23,42,43,45,46,47,48,50,51,52,53]
642	1.28	4-Ethyl-1,3-benzenediol (*m/z* 123, 138, 43, 67)	2896-60-8	3	1126	-	–
774	1.52	C_10_ Aromatic alcohol (*m/z* 115, 144, 63, 89)	-	3	1219	-	–
1332	1.93	2,6-Bis(1,1-dimethylethyl)-1,4-benzenediol	1020-31-1	2	1743	1683	–
		Cyclics					
846	1.65	1-Butyl-2-cyclohexen-1-ol (*m/z* 97, 39, 41, 69)	88116-46-5	3	1274	-	–
		Sulfur compounds					
78	0.33	Dimethyl sulfide	75-18-3	2	580	526	[52]
162	0.58	Dimethyl disulfide	624-92-0	2	727	731	[52]
330	1.02	1,3-Oxathiane	646-12-8	2	909	913	–
408	0.88	Dimethyl trisulfide	3658-80-8	2	963	969	[52]
438	2.86	3-(Methylthio)-1-propanol	505-10-2	2	985	989	[43,46,50,51,52]
456	0.74	5-Methyl-2-furanmethanethiol	59303-05-8	2	996	995	–
606	0.99	Ethyl 3-(methylthio)propanoate	13327-56-5	2	1101	1098	–
642	1.09	3-(Methylthio)-propyl acetate	16630-55-0	2	1126	1125	–
780	2.03	Benzothiazole	95-16-9	2	1224	1230	–
816	0.67	2-(1,1-Dimethylethyl)-thiophene	1689-78-7	2	1251	1251	–
		Volatile phenols					
588	2.89	Guaicol	90-05-1	1	1090	1095	[49]
858	2.01	4-Ethylguaiacol	2785-89-9	2	1283	1290	[50]
900	2.99	4-Vinylguaiacol	7786-61-0	2	1316	1333	[43,46,47,50,51]

^a^ Retention times for first (^1^*t*_R_) and second (^2^*t*_R_) dimensions in seconds. ^b^ Level of metabolite identification according to Sumner et al. [29]: (1) Identified compounds; (2) Putatively annotated compounds; (3) Putatively characterized compound classes; (4) Unknown compounds. ^c^ RI: Retention Index obtained through the modulated chromatogram. ^d^ RI, Retention Index reported in the literature for Equity-5 GC column or equivalents.

**Table 3:** Number and percentage of the common analytes within each chemical family, as well as the relative chromatographic area of the common and non-common analytes, considering the macro- and microbrewer beers.

Chemical Family	Number of Common Analytes	Number of Common Analytes within Each Chemical Family (%)	Chromatographic Area of the Common Analytes	Chromatographic Area of the Non-Common Analytes
Acids	10	58.8	>Microbrewer **	>Microbrewer *
Alcohols	37	61.7	Equal	>Microbrewer ***
Esters	64	73.4	>Microbrewer ***	>Microbrewer ***
Monoterpenic compounds	20	22.0	>Microbrewer ****	>Microbrewer ****
Norisoprenoids	9	56.3	Equal	>Microbrewer ***
Sesquiterpenic compounds	14	33.3	>Microbrewer ****	>Microbrewer ****
Sulfur compounds	10	76.9	>Macrobrewer ***	>Macrobrewer ***
Volatile phenols	3	100	Equal	–
TOTAL	167	50.8	>Microbrewer ****	>Microbrewer ****

Significant statistical differences are observed for *p* < 0.05 (*), *p* < 0.01 (**), *p* = 0.001 (***), *p* < 0.0001 (****), using *t*-test in GraphPad prism.

## Supplementary Materials

The following are available online at https://www.mdpi.com/2304-8158/9/9/1276/s1. Figure S1: Heatmap and dendrogram representation of 329 volatile components from lager beers under study. Figure S2: Box plots of the chromatographic area of acids for macrobrewer and microbrewer beers, considering the common (left) and non-common (right) analytes to all lager beers under study. Figure S3: Box plots of the chromatographic area of alcohols for macrobrewer and microbrewer beers, considering the common (left) and non-common (right) analytes to all lager beers under study. Figure S4: Box plots of the chromatographic area of esters for macrobrewer and microbrewer beers, considering the common (left) and non-common (right) analytes to all lager beers under study. Figure S5. Box plots of the chromatographic area of monoterpenic compounds for macrobrewer and microbrewer beers, considering the common (left) and non-common (right) analytes to all lager beers under study. Figure S6: Box plots of the chromatographic area of norisoprenoids for macrobrewer and microbrewer beers, considering the common (left) and non-common (right) analytes to all lager beers under study. Figure S7: Box plots of the chromatographic area of sesquiterpenic compounds for macrobrewer and microbrewer beers, considering the common (left) and non-common (right) analytes to all lager beers under study. Figure S8: Box plots of the chromatographic area of sulfur for macrobrewer and microbrewer beers, considering the common (left) and non-common (right) analytes to all lager beers under study. Figure S9: Box plots of the chromatographic area of sulfur for macrobrewer and microbrewer beers, considering the common (left) and non-common (right) analytes to all lager beers under study. Figure S10: Box plots of the total chromatographic area of all detected analytes for macrobrewer and microbrewer beers. Figure S11: Box plots of the short-chain (left, C_6_–C_12_) and long-chain (right, C_14_ and C_16_) esters for macrobrewer and microbrewer beers. Figure S12: Box plots of the chromatographic area of acetate esters for macrobrewer and microbrewer beers, considering the common (left) and non-common (right) analytes to all lager beers under study. Table S1: Full data set used for statistics processing including the detected metabolites in lager beers using HS-SPME/GC×GC-ToFMS, with relevant chromatographic data used to assess compounds identification.
