# Supplementary material for: Enlarging Knowledge on Lager Beer Volatile Metabolites Using Multidimensional Gas Chromatography
Source: Foods. 2020 Sep 11;9(9):1276. doi: 10.3390/foods9091276 (PMC7554861; doi:10.3390/foods9091276)
Supplement: Supplementary file 1 [file foods-09-01276-s001.zip › Supplementary information.pdf]

Enlarging knowledge on lager beer volatile metabolites using multidimensional gas chromatography

Cátia Martins [1], Tiago Brandão [2], Adelaide Almeida [3] and Sílvia M. Rocha [1,*]

[1] Departamento de Química & LAQV-REQUIMTE, Universidade de Aveiro, Campus Universitário Santiago, 3810-193 Aveiro, Portugal; catiamartins@ua.pt (C. M.)
[2] Super Bock Group, Rua do Mosteiro, 4465-703 Leça do Balio, Portugal; tiago.brandao@thebrowerscompany.com
[3] Departamento de Biologia & CESAM, Universidade de Aveiro, Campus Universitário Santiago, 3810-193 Aveiro, Portugal; aalmeida@ua.pt
* Correspondence: smrocha@ua.pt; Tel.: +351 234 401 524

Supplementary material

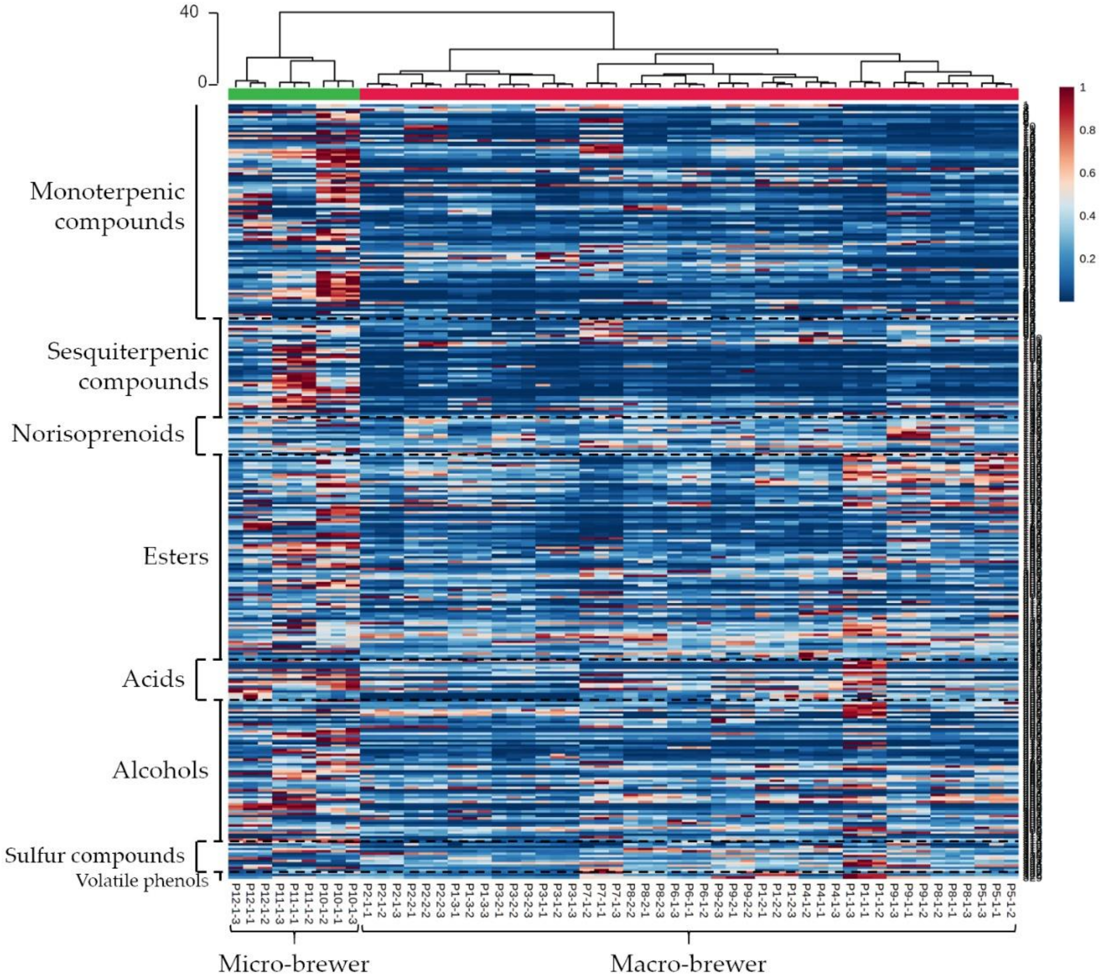

Figure S1. Heatmap and dendrogram representation of 329 volatile components from lager beers under study. The samples were clustered according to the producer' type: micro-brewer beers (green, obtained from Portuguese market); and macro-brewer beer (red, obtained from Portuguese market). The content of each compound was illustrated through different colors (from dark blue, minimum, to dark red, maximum). Dendrogram for the HCA results using Ward's cluster algorithm to the data set was also included.

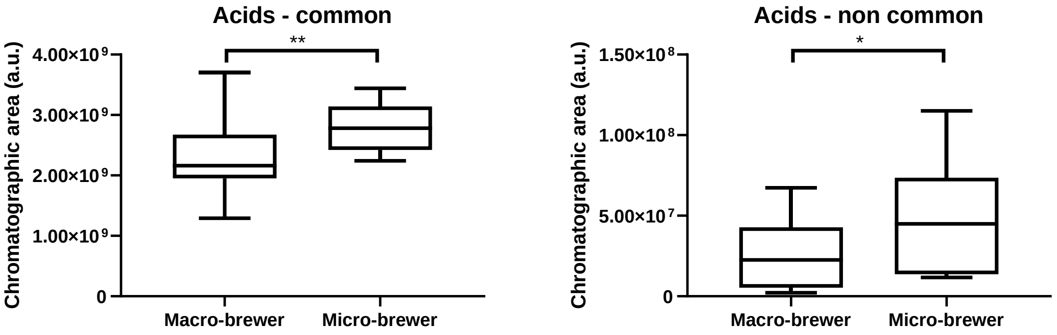

Figure S2. Box plots of the chromatographic area of acids for macro-brewer and micro-brewer beers, considering the common (left) and non-common (right) analytes to all lager beers under study. The beer producer type showed significant statistical differences, for both analytes (common and non-common), with $p < 0.01$ (**) and $p < 0.05$ (*) respectively, using *t*-test in GraphPad prism.

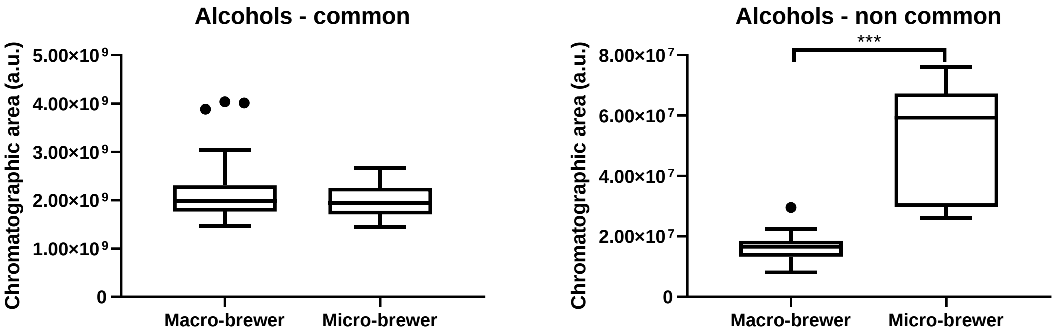

Figure S3. Box plots of the chromatographic area of alcohols for macro-brewer and micro-brewer beers, considering the common (left) and non-common (right) analytes to all lager beers under study. The beer producer type showed significant statistical differences for non-common analytes, with $p = 0.001$ (***), using *t*-test in GraphPad prism.

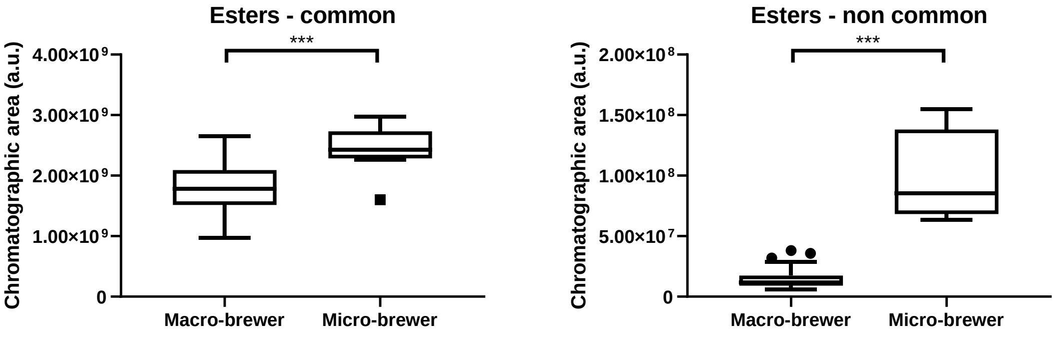

Figure S4. Box plots of the chromatographic area of esters for macro-brewer and micro-brewer beers, considering the common (left) and non-common (right) analytes to all lager beers under study. The beer producer type showed significant statistical differences, for both analytes (common and non-common), with $p = 0.001$ (***), using *t*-test in GraphPad prism.

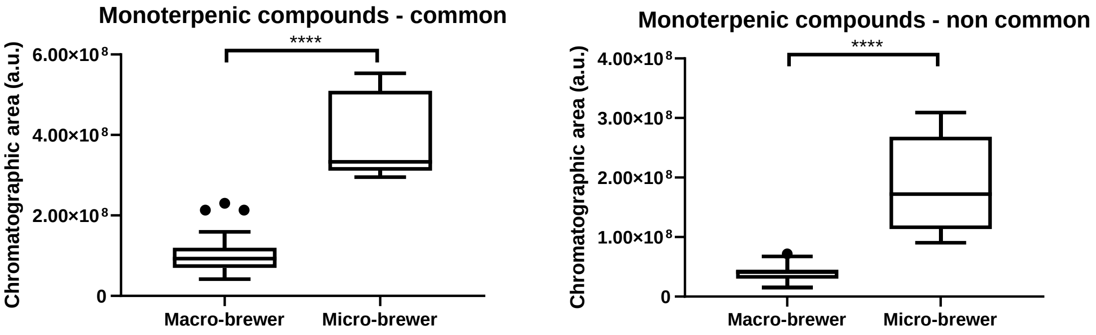

Figure S5. Box plots of the chromatographic area of monoterpenic compounds for macro-brewer and micro-brewer beers, considering the common (left) and non-common (right) analytes to all lager beers under study. The beer producer type showed significant statistical differences, for both analytes (common and non-common), with $p < 0.0001$ (****), using t-test in GraphPad prism.

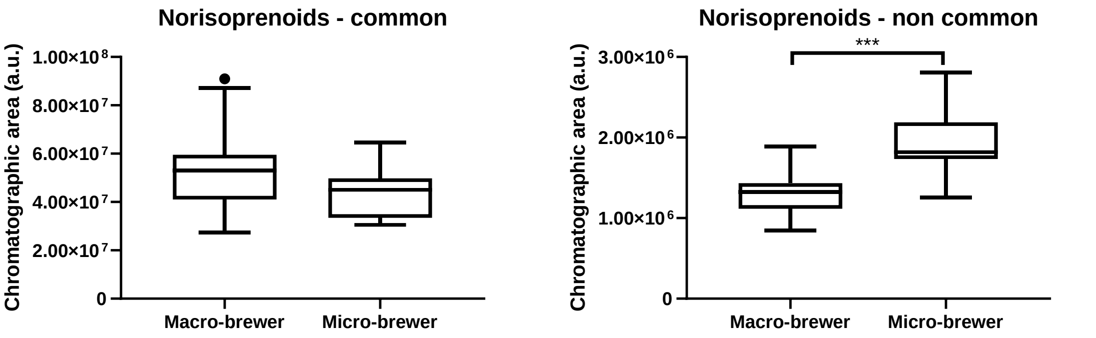

Figure S6. Box plots of the chromatographic area of norisoprenoids for macro-brewer and micro-brewer beers, considering the common (left) and non-common (right) analytes to all lager beers under study. The beer producer type showed significant statistical differences for non-common analytes, with $p = 0.001$ (***), using t-test in GraphPad prism.

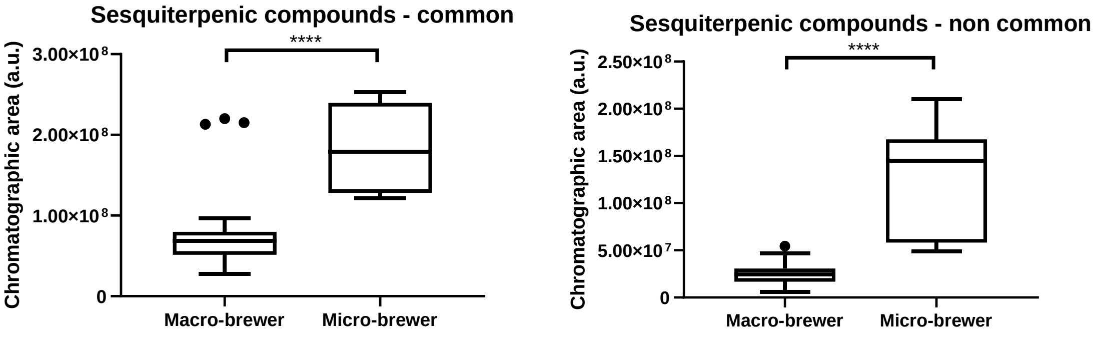

Figure S7. Box plots of the chromatographic area of sesquiterpenic compounds for macro-brewer and micro-brewer beers, considering the common (left) and non-common (right) analytes to all lager beers under study. The beer producer type showed significant statistical differences, for both analytes (common and non-common), with $p < 0.0001$ (****), using t-test in GraphPad prism.

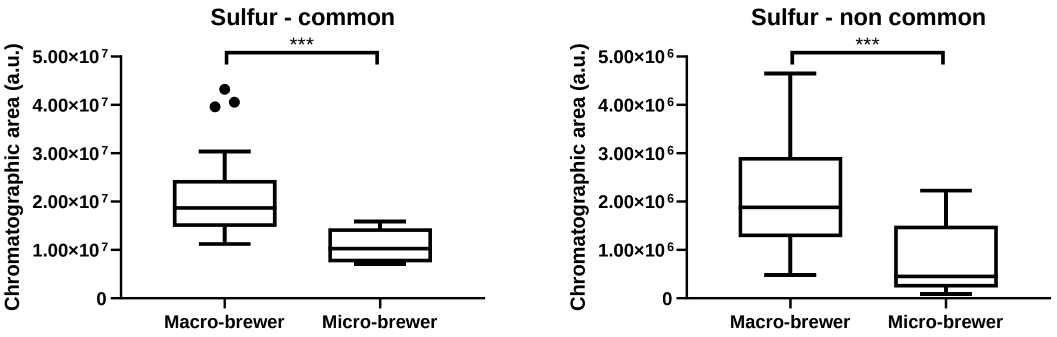

Figure S8. Box plots of the chromatographic area of sulfur for macro-brewer and micro-brewer beers, considering the common (left) and non-common (right) analytes to all lager beers under study. The beer producer type showed significant statistical differences, for both analytes (common and non-common), with $p = 0.001$ (***), using *t*-test in GraphPad prism.

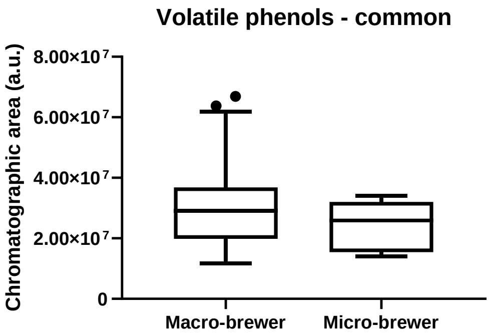

Figure S9. Box plots of the chromatographic area of sulfur for macro-brewer and micro-brewer beers, considering the common (left) and non-common (right) analytes to all lager beers under study.

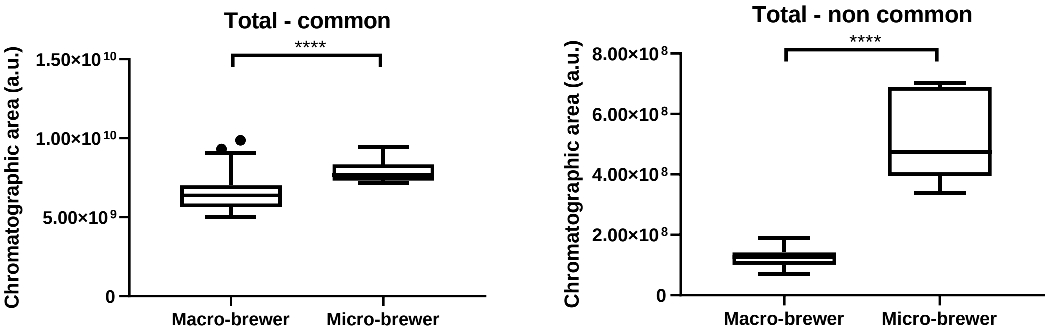

Figure S10. Box plots of the total chromatographic area of all detected analytes for macro-brewer and micro-brewer beers. The beer producer type showed significant statistical differences, for both analytes (common and non-common), with $p < 0.0001$ (****), using *t*-test in GraphPad prism.

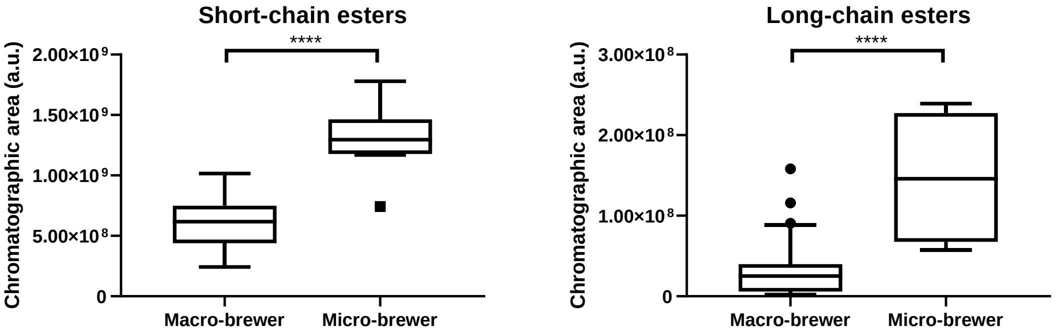

Figure S11. Box plots of the short-chain (left, C_6-C_{12}) and long-chain (right, C_{14} and C_{16}) esters for macro-brewer and micro-brewer beers. The beer producer type showed significant statistical differences, for both esters' type, with $p < 0.0001$ (****), using *t*-test in GraphPad prism.

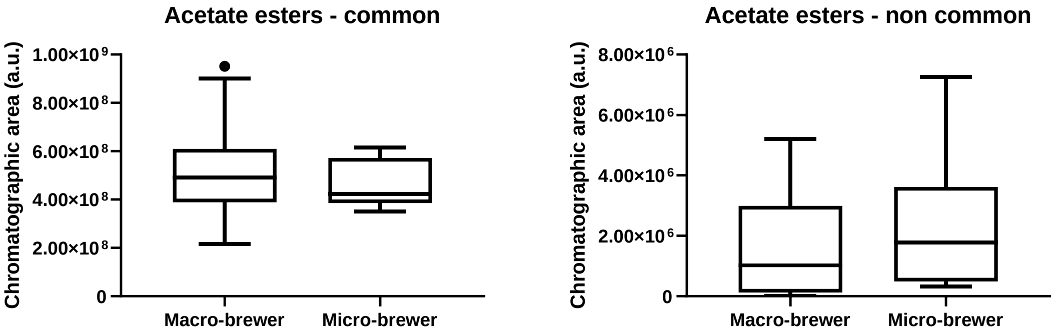

Figure S12. Box plots of the chromatographic area of acetate esters for macro-brewer and micro-brewer beers, considering the common (left) and non-common (right) analytes to all lager beers under study, which showed no significant statistical differences.
